# Top income shares in Canada: recent trends and policy implications

**DOI:** 10.1111/j.1540-5982.2012.01744.x

**Published:** 2012-11-20

**Authors:** Michael R Veall

**Affiliations:** Department of Economics, McMaster University

## Abstract

According to Canadian taxfiler data, over the last thirty years there has been a surge in the income shares of the top 1%, top 0.1% and top 0.01% of income recipients, even with longitudinal smoothing by individual using three- or five-year moving averages. Top shares fell in 2008 and 2009, but only by a fraction of the overall surge. Alberta, British Columbia, and Ontario have much more pronounced surges than other provinces. Part of the Canadian surge is likely attributable to U.S. factors, but a comprehensive explanation remains elusive. Even so, I draw implications for policies that might achieve some support from across the political spectrum, including the elimination of tax preferences that favour those with high incomes, the promotion of shareholder democracy and, to maintain Canada's relatively high intergenerational mobility, continued wide accessibility to healthcare and education.

## 1. Introduction

Research surveyed in Atkinson, Piketty, and Saez (2011) uses data derived from personal income tax filing to study the historical evolution of the top part of the income distribution for many countries. For Canada, Saez and Veall (2005, 2007) use such data to estimate the market income shares of the top 1%, 0.1%, and 0.01% of income recipients from 1920 to 2000. One of their findings, confirmed and extended by Murphy, Roberts, and Wolfson (2007) and Veall (2010), was that top shares surged in the last two decades of the 20th century.

**Figure 1:**
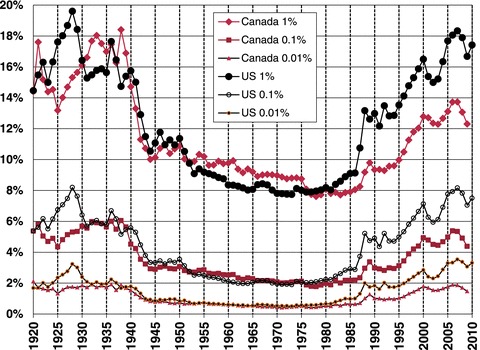
Income share of the top 1%, 0.1%, and 0.01%, Canada, 1920–2009; United States, 1920–2010 NOTE: Canadian results are by taxfiler; United States results are by family. SOURCE: Canada: author's calculations based on special order results provided to him by Statistics Canada using the Longitudinal Administrative Database; United States: Piketty and Saez (2003), as updated to 2010 at the website of Emmanuel Saez, http://elsa.berkeley.edu/~saez/TabFig2010.xls, March 2012, accessed 2 August 2012.

Figure 1, which contains new estimates that will be described in detail in section 2, shows that the surge did not continue smoothly after 2000, but that nonetheless Canadian top shares in 2009 were still markedly higher than they were in 1985.^1^,^2^ Section 2 also discusses the comparison with the United States, in particular arguing that comparisons of American and Canadian personal income tax data may overstate the difference in income concentration between the two countries. This section also discusses the income composition of the surge and shows that there is a surge in market pre-tax income with or without the inclusion of capital gains, and whether or not there is longitudinal smoothing by individuals using three- or five-year moving averages. The latter is important in ruling out one explanation for the surge: it is not simply a consequence of an increase in the variance of top incomes.

Sections 3 and 4 add new categories of estimates not provided by Saez and Veall on after-tax-and-transfer income and provincial trends respectively. Section 5 summarizes critically some of the explanations in the literature for the surge, finding no single explanation that is completely satisfactory. Section 6 discusses potential implications for taxation policy. Section 7 considers policies besides taxation. Section 8 is a brief conclusion.

## 2. The surge

Saez and Veall (2005) studied the 1920 to 2000 period and emphasize annual ‘market income,’ a definition which includes all income except government transfer payments and capital gains.^3^ This will also be the definition of income in this article, unless stated otherwise. Figure 1 includes the Saez and Veall estimates for Canada up to 1981 for the top 1%, top 0.1%, and top 0.01% income shares of individual filers as well as estimates from Piketty and Saez (2003) for the United States (by family) as updated to 2010 by Saez (2012). The Canadian observations for 1982 to 2009 are my new, updated estimates.

My new estimates for Canada have been calculated in a manner different from Saez and Veall in two respects. First, the new calculations are based entirely on the Longitudinal Administrative Database (LAD), a one-in-five anonymized taxfiler sample from the administrative data that are available from 1982. They involve no interpolation or extrapolation. (The Saez and Veall estimates were based on information by tax bracket. Top shares were estimated by Pareto interpolation/extrapolation and checked against the LAD data after 1982.) Second, the new estimates (and all estimates in this article, unless stated otherwise) are the shares of taxfilers;^4^ the earlier estimates were shares of adults aged 20 or above.^5^^,^^6^

Examining the recent Canadian estimates, we see that the surge in top income shares began in approximately 1985 and continued through to 2007.^7^ In 2008 and 2009, top income shares fell, as had occurred in previous recent recessions. While the United States surge began earlier than in Canada, recent patterns have been similar. Hence, the rise in the United States top shares in 2010 may predict higher Canadian top shares as well.

While the U.S. top share surge appears larger than the Canadian surge, one qualification should be noted. In the United States, a taxpayer (or small group of taxpayers) owning a business may choose two corporate structures for tax purposes. A C-corporation pays corporate taxes, and then any payments from the corporation to the individual are taxed through the personal income tax system, just as for Canadian-Controlled Private Corporations (CCPCs). An S-corporation pays no corporate tax: net revenues flow through directly and immediately to the personal tax return of the owner or owners. In the 1986 U.S. federal tax reform, there were corporation tax rate increases and other changes that led to a shift of income from C-corporations to S-corporations, which explains some of the U.S. surge (Gordon and Slemrod 2000). Hence, the true rise in U.S. top shares may have been overstated somewhat. By the same token, the levels of top shares in Canada are understated relative to the United States because some top share income is in effect hidden in the retained earnings of CCPCs.

**Figure 2:**
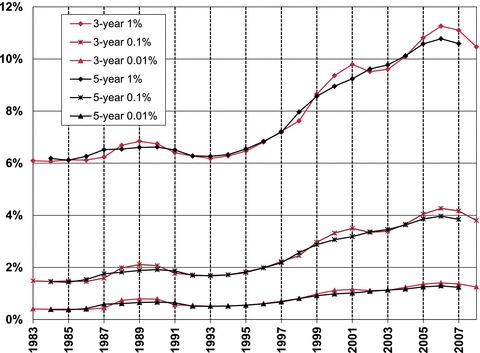
Income top shares, individual moving average, 1982–2009 NOTE: ‘Individual’ refers to ‘taxfiler.’ SOURCE: Author's calculations based on special order results provided to him by Statistics Canada using the Longitudinal Administrative Database.

Can the Canadian surge in annual top shares be explained empirically by a greater number of top-income individuals receiving large, serially uncorrelated bonuses? No. While paying bonuses may have become more common, figure 2 uses the longitudinal feature of the LAD by plotting three-year and five-year moving averages by individual. The figure still displays a substantial surge.^8^

What type of income was in the surge? Saez and Veall found that it was largely income declared for tax purposes as wage and salary income. Table 1 provides updated information that supports this view, showing that, comparing 1946 to 2009, the share of income that was capital income was roughly the same for the top 1% while falling for the top 0.1% and the top 0.01%. For all three of these top income categories, self-employment/business income fell and wage and salary income rose. However, it may be that what is employment income for tax purposes may be capital income from an economics perspective, most obviously in the case of an owner-managed firm.

**Table 1:** Shares of income as reported for taxes, Canada, 1946 and 2009

	Top 1%		Top 0.1%		Top 0.01%	
1946	2009	1946	2009	1946	2009
Wage income	45.5	64.9	34.0	63.3	27.2	64.8
Business income	34.4	13.4	32.4	8.7	19.9	1.5
Capital income	20.1	21.7	33.6	28.0	53.0	33.7

NOTES: Wages include wages, salaries, other employment income, and pensions. Business income is from self-employment and (unlimited) partnerships. Capital income includes dividend, interest, rental and other investment income, but not capital gains. 1946 value is from Saez and Veall (2007). Moving pension income from the wage category to capital income leads to only minor changes.

SOURCE: Author's calculations based on special order results provided to him by Statistics Canada using the Longitudinal Administrative Database.

## 3. Top shares of after-tax-and-transfer income

Table 2 uses LAD data to examine shares, thresholds, and levels of before-tax market income, excluding capital gains and after-tax-and-transfer income, including capital gains in 1986 and in 2009. Capital gains are included in the latter because the personal income taxes paid data in the LAD do not distinguish between taxes paid on capital gains and taxes paid on other kinds of income. The year 1986 is used because that was the first year in which the LAD data included three important types of untaxed transfer income: the Guaranteed Income Supplement, Workers’ Compensation, and Social Assistance. For the top 1% of after-tax-and-transfer income recipients, the 1986 to 2009 share increase from 7.1% to 9.9% is smaller than the increases for before-tax market income without capital gains from 8.0 to 12.3%. (While not reported in table 2, before-tax market income with capital gains increased from 9.0% to 13.3% over this period.) Continuing with after-tax-and-transfer income, table 2 shows that the top 0.1% and top 0.01% share surges in percentage terms are much larger than that for the top 1% share, and that the average real income of the top 0.01% increased by about 150% between 1986 and 2009, as opposed to an increase of 19% for those in the bottom nine deciles.^9^

**Table 2:** Top income recipients: shares, lower bounds, and averages, 1986 and 2009

	Before-tax market income, *excluding* capital gains			After-tax-and-transfer income, *including* capital gains		
1986	2009	% change	1986	2009	% change
*Percentage shares*						
P090	65.8	59.9	−9	70.20	67.10	−4
P9095	12.6	12.7	1	10.90	10.70	−2
P9599	13.6	15.1	11	11.80	12.30	4
Top 1%	8.0	12.3	53	7.10	9.90	38
Top 0.1%	2.2	4.4	100	2.00	3.60	80
Top 0.01%	0.6	1.5	132	0.60	1.21	105
*Lower bounds*						
P090	na	na	na	na	na	na
P9095	$70,300	81,600	16	$57,100	$68,600	20
P9599	$86,500	105,900	23	$69,300	$86,300	25
Top 1%	$143,900	206,900	44	$119,900	$162,300	35
Top 0.1%	$365,900	705,700	93	$300,600	$573,700	91
Top 0.01%	$1,061,600	2,694,600	154	$879,000	$2,137,200	143
*Average income within category*						
P090	$22,370	$24,100	8	$22,400	$26,500	19
P9095	$77,400	$92,100	19	$62,300	$76,200	22
P9599	$103,900	$136,200	31	$84,300	$109,200	30
Top 1%	$246,800	$444,800	80	$204,600	$352,000	72
Top 0.1%	$672,000	$1,581,300	135	$566,200	$1,268,100	124
Top 0.01%	$1,913,700	$5,284,000	176	$1,698,500	$4,297,500	153

NOTES: All dollar figures have been converted to 2011 dollars using the Consumer Price Index, all items. P090 corresponds to the bottom nine deciles. P9095 corresponds to those in the 91st, 92nd, … , 95th income percentiles. P9599 is defined similarly.

SOURCE: Author's calculations based on special order results provided to him by Statistics Canada using the Longitudinal Administrative Database.

## 4. Provincial trends in top shares

**Figure 3:**
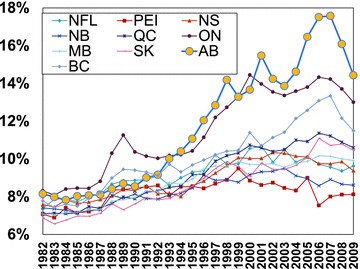
Top 1% income shares by province, 1982–2009 NOTE: Results are by taxfiler. SOURCE: Author's calculations based on special order results provided to him by Statistics Canada using the Longitudinal Administrative Database.

Again using market income excluding capital gains as the measure, figure 3 shows that the surge is much more pronounced in the provinces of Alberta, British Columbia, and Ontario than in the other provinces, Manitoba and the Atlantic provinces having the smallest surges. There will be further discussion of this later.

## 5. Potential explanations for the surge^10^

**Figure 4:**
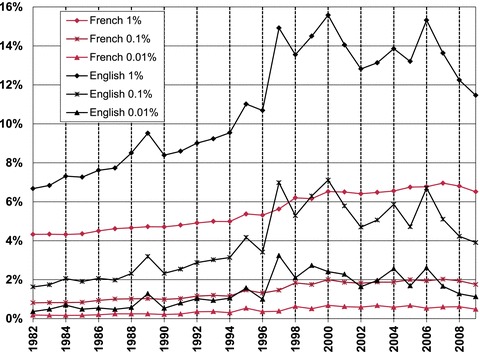
Top income shares in Quebec by filing language, 1982–2009 NOTE: Results are by taxfiler. SOURCE: Author's calculations based on special order results provided to him by Statistics Canada using the Longitudinal Administrative Database.

One explanation for the surge is ‘globalization’ (e.g., Krugman 2008). Increased international competition in the goods market may have reduced the demand for the relatively immobile labour involved in Canadian manufacturing, while at the same time there may have been increased mobility for some high-income workers to move to the United States. Figure 1 is consistent with Canadian changes in top shares being lagged responses to U.S. changes. Saez and Veall show that there is a much greater surge among residents of Quebec who file their personal income tax forms in English than for those who file in French, where possibly the former may be more affected by U.S. competition and perhaps U.S. corporate culture than the latter. Figure 4 uses the empirical approach adopted here along with more recent data to re-illustrate the Saez and Veall conjecture.^11^ However, cultural similarity may not be required, as Fabbri and Marin (2012) find evidence that German CEO pay is significantly affected by U.S. CEO pay. To the extent these findings suggest that the United States is the epicentre of the top share surge phenomenon, there would be the remaining question as to the cause of the surge in that country.

A second candidate explanation is skill-biased technical change (Katz and Murphy 1992) that may have disproportionately benefited those in high-income positions. The seminal paper of Sherwin Rosen (1981) explains generally how better technology, particularly communications technology, can magnify the returns to ‘superstars’ in any field, from entertainment to professional sports to management. For example, the theoretical study of Garicano and Rossi-Hansberg (2006) emphasizes the potential role of email and mobile technologies as they improve communications within the firm and hence increase the scope of those at the top of the firm to influence what happens lower in the hierarchy. Some research suggests that the ability to incorporate new technology has been particularly important in the financial sector (Philippon and Reshef 2009), which fits with the finding of Bakija, Cole, and Heim (2012) that increased incomes to financial professionals are a major component of the top income surge in the United States.

**Figure 5:**
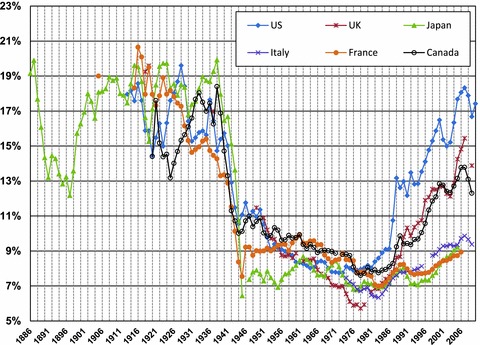
Top income shares by countries, 1886–2010 SOURCE: Alvaredo et al. (2012), The World Top Incomes Database, http://gmond.parisschoolofeconomics.eu/topincomes, World Top Incomes Database, http://g-mond.parisschoolofeconomics.eu/topincomes/ as accessed 17 May 2012.

A standard argument against the skill-biased technical change explanation for rising top shares is shown in figure 5, which uses the World Top Incomes Database and plots top income shares for the G-7 countries, except Germany, for which the comparable data are too limited. The surge from about 1980 to 2009 is clearly visible for the United States, the United Kingdom, and Canada. Clearly, there has been much less of a surge for Italy, France, and Japan. If the technical change explanation were complete, it might be expected that it would apply in all countries. While it may be possible to explain why the technology change had different effects on the income distributions of different countries and to link the timing of income distribution changes with the introduction of new technologies, the case currently remains unproven.^12^

A third type of explanation emphasizes executive compensation practices. One possibility within this type (e.g. Jensen and Meckling, 1976 and Jensen and Murphy, 1990) is that increased executive compensation can be an efficient consequence of an attempt to align top management salaries with those of shareholders. Gabaix and Landier (2008) emphasize the role of increasing firm size in explaining the increase in executive compensation, although Lemieux (2008, fn5) points out that the finding is sensitive to specification, and Gordon and Dew-Becker (2008) argue that it is sensitive to measures of firm size and choice of time period. A very different possibility is that of Bebchuk, Fried and Walker, 2002 and Bebchuk and Fried, 2004 who argue that higher CEO salaries are largely a result of the CEO's co-opting corporate governance by influencing the choice of company directors. Jensen and Murphy (2004) do not dismiss these concerns and indeed make a series of recommendations that might mitigate these effects including one that corporations ‘change the structural, social and psychological environment of the board so that directors (even those who fulfill the requirements of independence) no longer see themselves as effectively the employees of the CEO.’ However Jensen and Murphy (2004) maintain that these arguments do not explain what they believe is the over-use of options and the tendency for boards to pay more for CEOs hired externally. Bebchuk and Fried (2004) and Jensen and Murphy (2004) both emphasize that CEOs have strong incentives to control the information that determines their compensation.^13^^,^^14^^,^^15^ In a different but related context, use of insider information may be a particular concern in Canada, given the findings of Bris (2005), who, for a number of countries, examined increases in the prices of publicly traded equities in advance of the announcement of a takeover bid. Canadian prices tended to increase earlier than those in other countries, to a greater extent than in any other developed country.^16^

Finally, consider changes in taxation as a potential explanation, where in the following all references to taxation are to personal income taxation. Studies estimating the responsiveness of taxable income to changes in taxes now comprise a huge literature, founded in part on the seminal papers of Feldstein (1995) and the research in the volume edited by Slemrod (2000). One conclusion is that the compensated and uncompensated elasticities for hours of labour supply and total saving are likely quite low. However, for high-income individuals the elasticities with respect to taxable income are somewhat higher, perhaps because of all the decision margins that lie between the labour hours and saving decisions and reported taxable income, for example, effort, entrepreneurship, choice of residence, and particularly tax haven and tax planning decisions. The literature is summarized by Saez, Slemrod, and Giertz (2012) who focus on *e*, the elasticity of taxable income with respect to the net of tax rate (i.e., one minus the marginal tax rate). The overall conclusion is that *e* likely has a value for the United States between 0.1 and 0.4, higher values being more likely for those with high income. Even given the substantial reductions in marginal tax rates in the United States, it does not appear that these values would be sufficient to explain all of the surge in top incomes relative to average incomes in the United States.

There is evidence that Canadian tax responsiveness may be higher for high-income individuals. Sillamaa and Veall (2001) use data from 1986 to 1989 to study the effects of the tax changes in Canada in 1988 (as well as much more minor provincial changes). These changes included sharp reductions in top tax rates. They find a very large estimate of *e* of 1.67 for those who had 1986 gross incomes of $100,000 or more (roughly the top half of 1% of the income distribution), in sharp contrast to their estimate of approximately 0.25 for the entire population. However, they note the important caveat that their short time period may have caught largely intertemporal substitution and that the estimate is vulnerable to a secular trend in top incomes, as their method would tend to count any such trend as a behavioural response to the tax rate changes. There will be more discussion of this point below.

Gagné, Nadeau, and Vaillancourt (2004) use provincial aggregate data. For the 1988 to 1996 period and converting their tax elasticity estimates to net-of-tax elasticity estimates at a marginal tax rate of 0.5, their estimate for those with 1995 income of $150,000 or more (again fairly close to the top half of 1% of the income distribution) is even larger at 3. While it is not its main focus, Saez and Veall (2005) contains a relatively simple aggregate regression to estimate a top wage incomes value of *e* of 2.5 to 3 for the 1972 to 2000 period. When the trending variable the log of U.S. top 1% wage income share is included in the regressions, this range of estimates falls sharply to 0.18 to 0.28.

The Department of Finance (2010) uses two methods to estimate the tax sensitivity of high-income Canadians. Applying the method of Gruber and Saez (2002), individual data, and federal and provincial variation, their estimate of *e* for those with incomes $150,000 in 2006 dollars is 0.72. When the method of Saez (2004), the aggregate data, and federal and provincial variation in tax rates are applied, their estimate of *e* is 0.62.

The upper range of these estimates would be sufficient to explain the surge. Indeed the 2.5 to 3 estimate of Saez and Veall is essentially the answer to the question as to how big the elasticity would have to be if tax rates were the sole explanation. However, the bulk of the estimates are smaller.

A related issue is the imperfect timing. For example, figure 1 shows a blip in 1988 top shares associated with the top tax cuts that were the focus of Sillamaa and Veall, likely because top income recipients shifted their incomes intertemporally to take advantage of the lower tax rates. (This kind of intertemporal response is emphasized by Goolsbee (2000) in the U.S. context.) But the blip aside, figure 1 shows there was a trend of increasing top shares before 1988 and a continuing trend afterwards. Arguably the 1988 tax cuts are associated with a strengthening of that trend, but it is far from clear. In the case of Alberta (figure 3), the introduction of the flat tax in 2001, which cut top marginal rates in that province significantly, was followed by a sharp reduction in top shares in 2002 and 2003.

Hence, marginal tax rate cuts and the timing of the surge do not align perfectly. On balance, my tentative conclusion is that cuts in tax rates are part of the explanation for the surge in Canada, although I am uncertain as to how big a part because confounding factors, including potentially complicated lead and lag effects, make quantification of the relationship elusive. It may well be that much of the rest of the explanation centres on the United States for reasons that in my view are still undetermined.^17^

## 6. Potential policy implications of the surge for taxation policy

Without being able to pin down the cause of the surge, it is difficult to be definitive about its policy implications. However, there is likely to be significant policy interest. For example, according to a poll published by the National Post (Humphreys 2012), more than three-quarters of Canadians think that Canada suffers from an income gap, where the rich are getting too rich and the poor are getting too poor. Hence, this section will continue the discussion of taxation from the previous section, shifting to a policy focus. The following section will emphasize other potential policy implications of the surge.

A key question is whether tax rates on those with higher incomes should be raised, where again, unless stated otherwise, tax means personal income tax. Suppose the top end of the income distribution has the Pareto distribution and, for illustration, the goal is to raise as much tax revenue from top-income individuals as possible.^18^ Then, as in Diamond and Saez (2011), the maximum-revenue marginal tax rate for top earners is

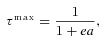
1where τ refers to a marginal tax rate and *e* is the elasticity of average top-end income with respect to the net of tax rate (x= one minus the tax rate). If an increase in tax rates reduces taxable income, *e* is positive. Intuitively, it is clear that the greater tax responsiveness, the less tax revenue will be raised for any increase in the tax rate and hence the lower the maximum-revenue marginal tax rate will be. The Pareto parameter *a* > 1 has the property that *r*=*a*/(*a*– 1) is the constant ratio of the average income above any threshold to the threshold itself. For example if *a* is 1.5, *a*/(*a*– 1) = 3 and the average income of all those with income above $500,000 will be $1.5 million and the average income of all those with income above $2 million will be $6 million.^19^^,^^20^

To make an obvious but perhaps not fully appreciated point, the surge in top incomes does not change the maximum-revenue marginal tax rate unless it changes *a* or *e*. Let us compare estimates of *a* (calculated directly from the empirical ratios *r* in the LAD) from 1989 (the year after the last major change in Canadian federal income tax rates), 2007 and 2009. The 1989 estimates of *a* are (1.98, 1.77, 1.79) for the top 1%, the top 0.1%, and the top 0.01%, respectively. For 2007, when top shares peaked, the corresponding estimates are (1.72, 1.71 and 1.95) and for 2009 they are (1.87, 1.81, 2.04). This suggests that *a* has not changed very much and is somewhere around two. Hence, if *e* has not fallen, and there are not strong reasons to believe it has, the recent surge in incomes would not imply an increase in the maximum-revenue top marginal tax rate. Of course it may be that actual top tax rates before the surge were not maximum revenue and that the implication of the surge is that the revenue gain that would come from increasing such rates is now much larger.

In any case, equation (1) and the ensuing discussion make clear the importance of the sensitivity of tax revenue *e* for the choice of marginal tax rates in any framework where the resulting tax revenue is a consideration. Table 3 explores this relationship by taking various values of *e* from the discussion that concluded the previous section. For each *e* and *a*, the table gives the revenue-maximizing top marginal tax rate and the actual revenue return to what would be a one dollar increase in taxes if there were no behavioural response and the initial tax marginal tax rate were 0.5. Diamond and Saez (2011) estimate *a* for the United States as 1.5. Given this and our estimates of *a* for Canada above, the table includes values of *a* in the 1.5 to 2.25 range. It can be seen that there is not huge sensitivity to the value of *a*, particularly compared with the sensitivity to *e*. Diamond and Saez use *e*= 0.25 as a ‘middle of the road value,’ given the survey of Saez, Slemrod, and Giertz (2012). With *a*= 2 and *e*= 0.25, the maximum marginal tax rate for top incomes is 0.67, and the behavioural response cuts the actual revenue increase from an increase in tax rates to about 50% of what it would be with no behavioural response. Given the two Department of Finance (2010) estimates of 0.62 and 0.72 and the Sillamaa and Veall (2001) estimates of 1.67, respectively, the maximum marginal tax rate is less than 0.5 and hence the actual return to a tax increase is negative.

**Table 3:** Top marginal rates and revenue from a ‘$1 tax increase’ on top earners as a function of selected values of *a* and *e*

	Top marginal rates				Revenue from a ‘$1 tax increase’			
*a*= 1.50	*a*= 1.75	*a*= 2.00	*a*= 2.25	*a*= 1.50	*a*= 1.75	*a*= 2.00	*a*= 2.25
*e*= 0.00	1.00	1.00	1.00	1.00	1.00	1.00	1.00	1.00
*e*= 0.15	0.82	0.79	0.77	0.75	0.78	0.74	0.70	0.66
*e*= 0.25	0.73	0.70	0.67	0.64	0.56	0.56	0.50	0.44
*e*= 0.40	0.63	0.59	0.56	0.53	0.40	0.30	0.20	0.10
*e*= 0.62	0.52	0.48	0.45	0.42	0.07	−0.08	−0.24	−0.39
*e*= 0.72	0.48	0.44	0.41	0.38	−0.08	−0.26	−0.44	−0.62
*e*= 1.00	0.40	0.36	0.33	0.31	−0.50	−0.75	−1.00	−1.25
*e*= 1.67	0.29	0.26	0.23	0.21	−1.50	−1.92	−2.34	−2.76

NOTES: Top marginal rates are based on equation (1). *a* is the Pareto parameter and *e* is the elasticity of taxable income with respect to the net of tax price. Both are discussed in the text. Revenue from a ‘$1 tax increase’= 1 –*ae* and is the actual increase in revenue from an increase in taxes that would raise taxes by $1 if there were no behavioural response, given an initial marginal tax rate of 0.5.

SOURCE: Author's calculations.

Hence, there is reason to be concerned that an increase in top marginal tax rates might not yield additional personal income tax revenue from highly paid individuals and might even reduce it. But there are at least three qualifications.

The first is that as noted, most of the econometric research has not included a U.S. variable in the specification. While the Saez and Veall (2005) analysis is not detailed, it is suggestive that in the one case where U.S. log shares are included as independent variables, the estimated value of *e* drops substantially to the 0.18 to 0.28 interval, which as table 3 notes, is in the range where top marginal tax rate increases will clearly increase revenue.

Second, it is sometimes argued that discussions such as these should include the effect that the higher tax rates may have on other tax-favoured and hence presumably desired behaviours (e.g., Chetty 2008). For example, higher tax rates may in some jurisdictions increase the incentive for higher charitable contributions. This argument extends imperfectly to Canada, given its wider use of tax credits and more limited use of deductions, unless a higher top tax rate automatically means more generous tax credits, in which case there is a direct loss of tax revenue from that change as well.

Third, Piketty, Saez, and Stantcheva (2011) consider a matching/bargaining model in which employee compensation increases with employee bargaining effort. Increases in the top marginal tax rate reduce the incentive to make bargaining effort and hence reduce the level of compensation.^21^^,^^22^ But because the bargaining is a zero-sum game, any reduction in that employee's income must accrue as income to someone else and be taxed. Expression (1) would not yield the marginal tax rate consistent with maximum revenue: in the most plausible case it would be too low.

The Piketty, Saez, and Stancheva approach decomposes *e* into a labour supply effect, a tax avoidance effect, and their bargaining effect. Given the labour supply effect is likely small, if the larger estimates of *e* for Canada are accurate, in their framework the difference must be tax avoidance response or bargaining response.

Pending research on the size and composition of *e* (which may come from studies of the Nova Scotia 2010 or the Ontario 2012 increase of top marginal tax rates), and, despite the qualifications noted, my own view is that there is some risk that increasing top marginal tax rates in Canada may yield only small or conceivably negative tax revenue gains.^23^ For those who advocate higher tax payments from those with high incomes, it may be more productive to concentrate immediate efforts toward the standard public finance prescription of broadening the tax base by eliminating special tax preferences, concentrating on those that differentially benefit those with high incomes.^24^ This approach potentially could find support from across the political spectrum.

The Department of Finance (2011) gives estimates of forgone tax revenues (‘tax expenditures’) associated with deductions, exemptions and tax credits in the personal income tax system. Some of these relate to the more favourable treatment of capital income as opposed to labour income. Many commentators think such special treatment is desirable for well-known reasons, even as they argue for a more efficient tax preference (see, e.g., Boadway 2011a).^25^

This is not the place for a deeper analysis of this question, which would lead to many issues such as inheritance taxes.^26^ However, regardless of the rate of capital income taxation, there are strong arguments against preferential treatment of different types of capital income. For example, Milligan (2005) and MacIntosh (2012) have been critical of the Registered Education Savings Plan deduction and the Labour Sponsored Venture Capital Corporation program, respectively. And concerning an issue in taxing employment income, there are strong arguments against the Employee Stock Option Deduction (Tedds, Sandler, and Compton 2012; Sandler 2001). In short I advocate a root-and-branch analysis of all tax preferences and the elimination of those that cannot be shown to contribute to the overall efficiency and the progressivity of the tax system.^27^ This will also be a step towards simplicity of the tax system.

## 7. Other policy implications^28^

With respect to taxation policy or other policy, the evidence of the surge itself does not necessarily call for a policy change. If a top-income surge is a requirement to retain talent or to align incentives correctly, then interventions to limit it might well not be helpful to the material interests of the majority of Canadians who are not top earners. On the other hand, there are legitimate concerns that income inequality may promote social division and concentration of political power^29^^,^^30^ in ways that most Canadians would find undesirable.

This sort of tradeoff is very difficult to evaluate. Instead, let me briefly outline two additional broad policy priorities that I favour, in part because I believe they also have some chance of support from across the political spectrum. It is not a coincidence that these policies, like the tax policy priority that I mentioned in the previous section, are plausibly productivity improving.

Of these the first is the area of corporate governance. As discussed earlier, it has been estimated that Canada has a relatively high prevalence of insider trading and it has not been immune to practices such as backdating options. Morck (2010) writes, ‘In practice, the typical big Canadian corporation is arguably less democratic than in the past, and less democratic than its peers in both America and Great Britain. This is because corporate insiders dominate the shareholder meetings of listed Canadian firms to an extent generally not seen in either the United States or the United Kingdom, and because Canadian legislatures, courts, regulators, and exchanges accept and passively perpetuate this.’ An environment of insider control seems likely to foster excessive CEO compensation, in which case high compensation may be a symptom of something far worse, as Morck continues, ‘a large and growing body of evidence shows Canadian corporations underperforming across the board’ and that this is ‘no coincidence, for much empirical evidence links shareholder democracy to firm and economy performance.’

Therefore ‘say on pay’ laws, where shareholders must approve CEO compensation packages (as in, e.g., Australia and the United States) or be given an opportunity for a non-binding vote (as in, e.g., the United Kingdom and Germany), are unlikely to be sufficient. In any case a number of Canadian corporations are voluntarily adopting such measures (perhaps thereby increasing their share prices; see Trottier 2011). Morck argues for the reduction of the power of controlling shareholders through measures to make non-voting shares and pyramiding^31^ less attractive, and to ensure the independence of pension fund trustees. He also supports national securities regulation to prevent a race to the bottom among provincial securities regulators. Policies that limit the power of insiders (on all matters, but including executive compensation) can aid the raising of capital (by acting as commitment devices for the raisers) as well as promoting a more vigorous market for corporate control and hence better management, capital allocation and growth.^32^

On my second non-tax broad policy priority, many would argue that one of the most negative aspects of inequality is intergenerational immobility. If a high-ability child born to lower socioeconomic status has little chance to advance and use her or his talents, or if someone of low ability takes home a large salary as the CEO of the family-controlled firm, it may be widely seen as unfair but it will also lead to a less dynamic and productive economy.

**Figure 6:**
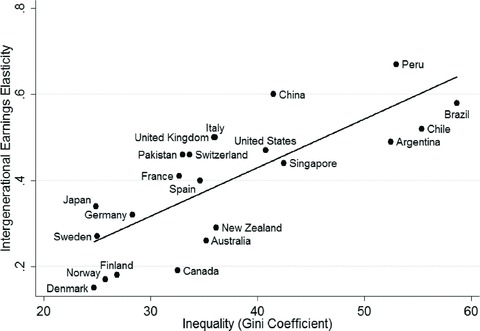
The relationship between earnings inequality and intergenerational earnings mobility across countries SOURCE: Corak (2012)

One summary measure of mobility is the intergenerational transmission elasticity, which is most often computed as the elasticity of son's income with respect to father's income, calculated at appropriate points in time. A low value corresponds to high mobility. Corak and Heisz (1999) estimate this value for Canada at about 0.2, and similar estimates are obtained by Fortin and Lefebvre (1998). Figure 6, taken from Corak (2012), puts this in the context of estimates from other countries and notes that even though Canada has much higher inequality (i.e., a higher Gini coefficient) than the Scandinavian countries, its intergenerational mobility is almost as high. Intergenerational mobility is also much higher than in other countries with similar Gini coefficients (e.g., France, the United Kingdom) or in countries with much higher Gini coefficients (e.g., the United States).^33^

The relatively high level of Canadian mobility is most likely attributable to the public availability of healthcare and the education system (see, e.g., the striking results of Currie 2012 regarding the importance of prenatal and early childhood care; Corak, Curtis, and Phipps 2011 for evidence on the differences between child outcomes in Canada and the United States; Davies, Zeng, and Zhang 2005 for a theoretical treatment of the inequality-reducing effects of education). Corak (2012) notes that an important difference between Canada and the United States is that Canadian students from families of lower socioeconomic status are relatively more likely to receive a high-quality education in primary and secondary schools. This in turn improves their access to post-secondary education.^34^ As healthcare accessibility and education accessibility are largely under the jurisdiction of provinces in Canada, and the budgetary situation of a number of provinces is increasingly dire, such policies may be at increasing risk and hence inequality of opportunity may rise.^35^

## 8. Conclusions

The surge in top share incomes in Canada over the last 30 years is clear. It appears plausible that the Canadian surge is a reflection of a bigger U.S. surge, although the relationship may differ across industry and sector, given the concentration of the Canadian surge in Ontario, Alberta, and British Columbia. I find no single explanation of the surge in either country to be completely satisfactory, but I do suggest that some of the surge is likely a consequence of a principal-agent problem in the relationship of shareholders and CEOs/managers. If this were true, it might well be that the pay surge is a symptom of more serious allocation problems, manifested in part by the economic crisis of 2008, which continues at time of writing.

Without knowing the cause of the surge, policy recommendations must be qualified. Given that, I suggest that those concerned about inequality should target three policy priorities. These priorities are related positively to productivity and, perhaps because of that, may well receive support from across the political spectrum.

First, with respect to taxation, my review of research on tax responsiveness in Canada leads me to believe that, given current knowledge, there is some risk that increases in the top marginal tax rates might raise little or no revenue. If the goal is to increase taxes on those with high incomes, I would argue that the immediate priority should instead be broadening the personal income tax base, particularly eliminating tax preferences that are likely to be taken advantage of by the upper end of the income distribution. I encourage ‘root and branch’ research on the effectiveness of these preferences and cite as examples the research of Milligan (2005) on Registered Education Savings Plans, MacIntosh (2012) on Labour Sponsored Venture Capital Corporations, and Tedds, Sandler, and Compton (2012) and Sandler (2001) on the tax treatment of stock options.

A second policy priority should be corporate governance. Morck (2010) makes a convincing case that shareholder democracy is too weak in Canada. In line with my topic, I note that excessive insider power may lead to inappropriately high executive compensation, but if Morck is correct, this is not the most important consequence of a much more serious malaise. Morck outlines a number of policy directions to limit insider power in ways that he argues would improve capital markets and the market for corporate control, enhancing Canadian economic performance.

The third policy priority concerns intergenerational mobility, which Corak and Heisz (1999) estimate for Canada. Corak (2012) shows that, while Canadian estimated income inequality is not particular low internationally, Canadian estimated intergenerational mobility is particularly high. This seems most plausibly linked to the healthcare accessibility and particularly educational accessibility policies of Canadian provinces. Given the likely fiscal threats faced by a number of provinces in the upcoming years, maintaining the accessibility required to prevent a rise in inequality of opportunity will be a substantial policy challenge.

## Footnotes

1 This article situates in the literature on the Canadian income distribution, which is too extensive to survey here. However, the pioneering study of Goldberg and Poduluk (1957) found evidence of decreasing inequality from 1930 to 1950 (based on census wages and salaries data). Heisz, Jackson, and Picot (2001) survey some of the more recent literature, including the important study of Beach and Slotsve (1996). This literature mostly used the more complete census income data available since the 1971 census and finds some evidence of increasing inequality in the 1980s and 1990s.

2 The surge is concentrated at the top. Using the data set that underlies figure 1, I estimate that the market income share (not including capital gains) of the top decile in Canada (not graphed in figure 1) rose from 34.2% in 1986 to 40.1% in 2009, an increase of 5.9 percentage points. More than 70% of this 5.9 is accounted for by the share increase for the top 1% from 8.0 to 12.3%.

3 Capital gains should arguably not be thought of as part of annual income in the year they are realized because they represent income earned over a number of years. In any case, capital gains were not taxable in Canada before 1972 and hence were not captured by taxfiler data before that year.

4 In Canada, personal income tax filing is by individual, but couples can largely be identified in the LAD data. ‘Filers’ include a small number of spouses with very low income who do not file individually but whose information is included in the return of their spouse. In the United States most couples file jointly. Accordingly, Piketty and Saez work at the couple level, but make adjustments for non-filers. Saez and Veall (2005) find only small differences between the results for individual filers and those for couples.

5 As a consequence, the splice of the series at 1982 is imperfect. For the top 1% series, the Saez and Veall series exceeds the new series by about half a percentage point for 1982 and for 2000, although for most of the intervening years the gap is very small. There is much closer agreement throughout for the top 0.1% share series and the top 0.01% series. Regardless, there has clearly been a surge in top incomes and hence these splicing issues will be ignored in this paper.

6 In both Saez and Veall and here, there is no adjustment for changes in the age composition of the population. Veall (2009) finds a larger surge when the analysis is restricted to the older population.

7 Osberg (2011) emphasizes that the surge is in the numerator of the top shares; that is, it is an increase in the absolute real incomes of those at the top end of the distribution.

8 In addition, the empirical transition probabilities of leaving the top 1%, 0.1%, and 0.01% were 0.32, 0.46, and 0.55, respectively, for 1985 to 1986 but were lower at 0.30, 0.40 and 0.54 from 2008 to 2009. Thus, if anything, within category persistence has increased over time. Beach (2006) and Beach, Finnie, and Gray (2010) have found that overall earnings mobility has been falling in Canada over this period.

9 When the LAD is used to construct couples and census families, the after-tax-and-transfer income surge is very similar for couples or for adult equivalents, where the family size adjustment is the square root of the number of family members.

10 The useful summary of Bakija, Cole, and Heim (2012) has influenced this discussion, although my taxonomy is somewhat different.

11 The differences across provinces of figure 3 might be seen as less supportive of the hypothesis that the Canadian surge is U.S. driven, although perhaps the financial industries of Ontario and British Columbia and the oil/gas industry of Alberta have particularly strong U.S. links.

12 There were other countries with clear surges, including Australia, New Zealand, and Ireland, where perhaps it is important that these countries have a legal system with British roots or that they are English-speaking and hence are closer culturally to the United States. (On the latter point, see the previous discussion of globalization and different trends for English and French speakers in Quebec.) There are other countries where the evidence suggests very small surges, for example, Spain, Switzerland, Sweden, and Denmark. The German case is complicated, but it appears as if top share inequality and wage inequality trends may be different. The data in the World Top Incomes Database are from Dell (2007), are based on taxfiler data, and end in 1998. No surge is reported for the top 1%, top 0.1%, or top 0.01% shares in the 1980s and 1990s. Bach, Corneo, and Steiner (2009) report similar results up to 2003, but find a surge in the top 0.001% and 0.0001% shares. However, over roughly this same period, Fabbri and Marin (2012) find increasing CEO salaries using executive compensation data, and Dustmann, Ludsteck, and Schonberg (2009) find increasing wage inequality using (right-censored) social security data.

13 Martin (2011) and Brooks and McQuaig (2010) also argue against methods currently used to determine CEO compensation, the latter strongly maintaining it is excessive in both Canada and the United States. Frydman and Saks (2010), who examine U.S. CEO compensation directly, argue that there is no corporate governance explanation that lines up well with the timing of the U.S. surge. However, while speculative, one possibility might be the technological developments that allowed a more liquid options market; for example, the Chicago Board Options Exchange opened for a limited number of stock call options in 1973.

14 Relatedly, a common explanation of the crisis of 2008 is that subordinate financial managers in some financial entities in the United States were rewarded for increasing the valuations of assets in their accounts, with insufficient adjustment for risk. As the values of the accounts of individual managers were aggregated as part of the valuation of the firms themselves, CEOs rewarded by such valuations had a disincentive to question the underreporting of risk or the acquisition of more risk. From this viewpoint, what happened next is well captured by the aphorism attributed to John Kenneth Galbraith: ‘Recessions catch what auditors miss.’ A very different explanation of the crisis also related to top share inequality is that of Kumhof and Rancière (2010). In their model the desire of those with high incomes to save can only be matched by lending to those with low incomes who eventually take on more than they can repay, leading to collapse of the financial system.

15 Piketty and Saez (2006) suggest that cultural explanations may go beyond corporate culture. For example there may be a constraint as to what level of compensation may be socially acceptable.

16 McNally and Smith (2003) reported poor disclosure of insider trading at the Toronto Stock Exchange with McNally and Smith (2010) reporting marked improvement. Compton, Sandler and Tedds (2009) raise serious issues regarding options backdating in Canada which Compton, Nicholls, Sandler and Tedds (2012) study further in a taxation context. Tedds, Compton, Morrison, Nicholls and Sandler (2011) find shortcomings in public disclosure of granted options in Canada.

17 As noted, Saez, Slemrod, and Giertz (2012) conclude that tax responsiveness among U.S. top-income recipients is not high enough for the U.S. surge to be caused solely by tax changes. However, Piketty, Saez, and Stantcheva (2011) point out that there is a correlation between tax reductions for top-income recipients in many countries and increases in top shares, although there are exceptions. For example, note from figure 5 that Japan and Italy have had only small top income share surges, even though those countries have had substantial cuts in top marginal tax rates.

18 From a perspective of positive economics, this would be the implication of the median voter model as applied, for example, by Acemoglu and Robinson (2006). While I believe it would be a common view that tax rates for those with top incomes should never be higher than the point of maximum revenue extraction, some might maintain that the reduction in inequality from still higher tax rates would be an advantage that would offset the loss in taxation revenue.

19 To help understand intuitively the role of the Pareto parameter in (1), consider that an incremental reduction in the marginal tax rate applied to the top bracket will have two theoretical effects. The first effect is to stimulate earnings and hence increase tax revenues (assuming strictly positive *e* throughout). The second effect will decrease tax revenues by cutting the tax rate on income that would have been earned without the tax reduction. For a larger Pareto parameter *a*, the mass of income is closer to the lower limit of the tax bracket, because *a/*(*a*− 1) becomes closer to one, and hence there is less income subject to the second effect, leading to a lower optimal marginal tax rate.

20 Saez (2001) also considers optimal marginal tax rates at other levels using this elasticity approach. Boadway (2011b) provides a survey of the theory of redistribution policy with implications for practice.

21 The standard approach in this literature implicitly assumes a supply and demand model for high-income workers with perfectly elastic labour demand. Hence, increasing the tax rate does not change the pre-tax wage. If the model is modified to make labour demand not perfectly elastic, increasing the tax rate increases the pre-tax wage. This contrasts with the Piketty, Saez, and Stantcheva model, where increasing the tax rate will reduce the pre-tax wage.

22 From one of their examples, consider that the outside option for an employee involves moving costs (e.g., switching houses, changing the children's schools, costs associated with a spouse finding a new job). The value of the option is the after-tax gain less these costs and hence falls as tax rates increase. Hence, tax rate increases weaken the employee's bargaining position and dampen the employee's salary.

23 Top marginal tax rates in most provinces currently exceed 50%, given a reasonable allowance for consumption taxes. (Formal inclusion of consumption taxes in the analysis would be complex, for example, because some expenditures are untaxed and different taxes have different avoidance possibilities.)

24 There would remain the choice as to whether to use the extra tax revenue produced to reduce government debt, to lower tax rates (and if so for which group) or to raise government transfers or spending. In the context of the discussion here, reducing tax avoidance opportunities will reduce *e* and hence increase the revenue gains from increasing the top marginal tax rate. Still, any increase in progressivity from removing tax preferences alone is likely to be limited, unless those changes involve the taxation of capital income or inheritance income.

25 One difficulty is that savings plans accounts, such as Registered Retirement Saving Plans (RRSPs), may not affect the marginal after-tax returns of large savers because they hit the contribution limit. Indeed the present-value approach in the Department of Finance approach to estimating the tax expenditures associated with RRSPs and Registered Pension Plans (RPPs), which I nonetheless argue is more useful than the cash flow approach also presented, assumes that RRSPs and RPPs do not affect saving behaviour. To the extent that they do stimulate saving, the tax expenditures are overestimated (Robbins and Veall 2002).

26 It would also lead to discussion of wealth inequality. Murphy, Roberts, and Wolfson (2007) found for Canada that top wealth shares tend to be higher than top income shares and at the top end a greater percentage of wealth is financial wealth. More recently, Davies, Lluberas, and Shorrocks (2011) estimate that the top 1% wealth share in Canada is about 24.0%. Their estimates for France and Germany are slightly higher at 25.2% and 25.5%, respectively, and much higher for the United States at 36.8%. Their estimates for the remaining G-7 countries are lower than for Canada with 21.4% for the United Kingdom and 17.4% for each of Italy and Japan. For Canada in 1970, Davies (1979) estimates the top 1% wealth share as about 20%.

27 In passing, I support the call of Boadway (2011, 185) for tax refundability of most tax credits. For example, while I do not see a strong prima facie case for either the Children's Art Tax Credit or the Children's Fitness Tax Credit, if they are to exist, I find it even harder to see the case for their being non-refundable as is currently the case. In effect, these subsidize the participation in the arts and sports activities for children in all families except those too poor to be subject to personal income tax, probably the only families for which the subsidy might make an appreciable difference. If it is judged that refundability of a tax credit is too expensive, my view would be that the rate of the tax credit should be lowered until refundability is affordable.

28 Fortin et al. (2012) also provide recommendations regarding inequality, consistent with the view of tax policy that I present, but also emphasizing labour market policies. They also make the important observation that the top 1% includes many in non-managerial occupations such as doctors and lawyers, whose compensation increases may not be well explained by the possibilities in section 5.

29 As an example of a consequence of income and wealth polarization in the United States, Drutman and Phelps-Goodman (2012) calculate that for the United States the top 0.01% of donors made close to 25% of all contributions to political campaigns. Hacker and Pierson (2010) argue that the U.S. surge is rooted in a more effective use of lobbying and campaign funding by business interests, beginning in the late 1970s. Acemoglu and Robinson (2012) argue that there is the potential for a vicious circle if increasing wealth enables the wealthy to influence policy in ways that favour the wealthy.

30 Largely focusing on the United States, Stiglitz (2012) argues that, along with agency problems in corporate governance, much of the increase in top shares is due to successful rent-seeking within the political process, at the expense of economic growth.

31 ‘Pyramiding’ is the practice of a firm holding a controlling interest in a number of other firms, which in turn can hold controlling interests in other firms, and so on. It can concentrate corporate power in the hands of a few. Morck explains that tax and other laws essentially have eliminated this practice in the United States and the United Kingdom, although it is common elsewhere.

32 While in my view government intervention in the actions of a corporation should be kept to the minimum, government policies that mandate accountability and transparency are akin to consumer-protection regulation in that they can reduce agency problems and hence be of value to all market participants.

33 Leigh (2007) shows that top shares and Gini coefficients are highly correlated across countries.

34 This is not to argue that the top-share phenomenon is largely a consequence of returns to education but that mobility up the income distribution, including to the very top, may be facilitated by good-quality education.

35 Such policies also may be increasingly important. Corak and Heisz (1999) estimate that, for fathers in the top 1% of income recipients, the intergenerational transmission elasticity increases to about 0.4. Björklund, Roine, and Waldenström (2008) are able to examine a sample from the top 0.1% in Sweden and estimate an intergenerational transmission elasticity of over 0.8. Besides providing more recent estimates that confirm the Corak and Heisz findings, Corak and Piraino (2010, 2011) find that the sons of fathers in the top 1% of the earnings distribution have a more than 15% empirical probability of having the same main (private sector) employer as their father, about twice as high as for sons of fathers who were in the 95th percentile and compared to about 6% for all sons. Hence, it may be that the rise in top- share incomes in Canada presages a reduction in intergenerational mobility.
